# Understanding consumers’ purchase intentions of single-use plastic products

**DOI:** 10.3389/fpsyg.2023.1105959

**Published:** 2023-02-21

**Authors:** Ying Sun, Haonan He

**Affiliations:** ^1^School of International Economics and Management, Beijing Technology and Business University, Beijing, China; ^2^School of Economics and Management, Chang’An University, Xi'an, China

**Keywords:** theory of planned behavior, normative social influence, informational social influence, positive anticipated emotion, intention to purchase single-use plastic products

## Abstract

Human health and marine life are facing the hazards and threats of plastic waste. China is the world’s largest producer and consumer of disposable plastic products, thus paying more attention to the threats and challenges of single-use plastics products in China is urgent. This study aims to explore the intention to purchase single-use plastic products based on the theory of planned behavior. Data collection using self-reported questionnaires, and 402 valid questionnaires were obtained, thus analyzed using Amos 22.0 and SPSS 18.0 software. Results indicate that attitude, perceived behavioral control, normative social influence, informational social influence, and positive anticipated emotion positively affect intention to purchase single-use plastic products. Meanwhile, positive anticipated emotion positively moderates the relationship between normative social influence and intention to purchase single-use plastic products, but negatively moderates the relationship between informational social influence and intention to purchase single-use plastic products. This research provides some theoretical and policy implications to help relevant agencies design targeted interventions to address environmental issues related to single-use plastic consumption.

## 1. Introduction

Borrelle et al. (2020) indicate that up to 23 million tons of plastic waste enter the environment each year. It is urgent for all parties to jointly explore global plastic pollution control methods and jointly protect their homes (Kautish et al., 2021; Luo et al., 2021). Making plastic products requires a lot of energy. They are composed of petroleum-derived substances and may take up to 200 years to degrade. In addition, the vast majority of single-use plastic products, such as plastic bags, are discarded as waste after one use, and their average usage time does not exceed 20 min (Zambrano-Monserrate and Ruano, 2020; Borg et al., 2022). After entering the natural environment, plastic products are difficult to be broken down by sunlight or microorganisms. Although most countries have issued plastic ban orders, plastic pollution is still regarded as an environmental disaster, threatening the survival and development of human beings. In this century, oceans all over the world are affected by plastic garbage, and there are approximately 46,000 pieces of plastic per square kilometer. These figures are alarming, and if the pattern of consumption and waste management does not change, about 12 billion tons of plastic waste will be generated by 2050 (UN, 2018).

Single-use plastic products are a general term for plastic products for production and life that are processed with plastic as the main raw material and are not intended for repeated use (Chen et al., 2021). They are widely used around the world because of their light weight and corrosion resistance. China is the world’s largest producer and consumer of disposable plastic products (Wang and Li, 2021). The *per capita* consumption of disposable plastic products exceeds 25 kilograms per year, which is twice the global average. China faces challenges in regulating plastic production, consumption, and back-end processing. The situation is more serious. Therefore, it is more urgent to pay attention to the purchase and use behavior of single-use plastic products in the context of China. Research on the purchase of single-use plastic products in China has gradually increased, but is still relatively small.

Consumers play a vital role since they are the ultimate driver of plastic production and consumption. By guiding consumers’ consumption behavior, it can effectively decrease the proportion of sustainable, environmentally friendly, and socially responsible consumption, and provide solutions for reducing and eliminating plastic pollution (Zambrano-Monserrate and Ruano, 2020). Previous scholars have carried out preliminary explorations on the determinants of consumers’ intention to purchase single-use plastic products. The existing literature on consumer behavior to solve the problem of plastic pollution mostly focuses on cognitive factors, such as consumers’ awareness of plastic pollution, and attitudes toward environmentally friendly behaviors (Ohtomo and Ohnuma, 2014; Zambrano-Monserrate and Ruano, 2020; Pham et al., 2021).

According to Montano and Kasprzyk (2015), academics have used various models to study consumers’ environmental behavior. Norm activation model (NAM), theory of reasoned action (TRA) and theory of planned behavior theory (TPB) are all widely used frameworks (Ajzen, 1991). However, because NAM ignores external factors and only focuses on internal factors, it has been criticized by scholars (Shi et al., 2017). TRA is used to predict the intentions and behaviors of individuals in daily life, but because the assumptions of the model are perfectly rational, it has also been criticized by many researchers. Compared with previous theories, TPB takes into account both non-volitional factors and external factors, such as perceived behavioral control in non-volitional factors and subjective norm in external factors. Therefore, it is reasonable to choose TPB as the basic theory of this study to explore consumers’ purchase intention behavior for single-use plastic products (Wang et al., 2020; Aruta, 2022). Moreover, many researchers believe that in order to improve the explanatory power of the TPB model, additional factors can be added according to different situations (Shi et al., 2017; Sun et al., 2021).

In addition, consumers are not always rational in their decision-making process, they often mix the influence of emotions in their decision-making (Wang et al., 2018). Emotions are not only an important way for us to express feelings, but also information, special information that characterizes various complex social relationships (Armon-Jones, 1986; Sui and Li, 2020). In interpersonal communication, people often express their emotions, but also need to interpret the emotions of others. Individual emotions not only affect themselves, but also affect others, so emotions have social effects. Hence, under the discussion of opposing the environment-friendly behavior of buying single-use plastic products, consumers’ subjective norms will also be affected by consumers’ emotions. From this perspective, emotions are also social, and a complete explanation of their adaptive utility needs to understand their mutual influence on interaction partners. Therefore, we study consumers’ purchase behaviors of single-use plastic products from the perspective of social research, and also introduce the variable of consumer emotion.

This study considers the decision-making process of cognitive and emotional factors in the purchase and purchase behaviors of single-use plastic products. Overall, this research has some contributions. Firstly, this research considered emotional factors besides cognitive factors, and also considered the interaction with emotional factors in the context of social research, which enriched the application of planned behavior theory in the context of purchasing single-use plastic products. Emotional factors and their interaction with subjective norms are combined into TPB to understand consumers’ pro-environment behavior, which enriches the understanding of consumers’ behavior of not purchasing single-use plastic products. Secondly, the rationality of TPB in the field of green behavior has been verified. It is proposed that attitude, subjective norms, and perceived behavioral control are directly proportional to consumers’ intentions. However, the three variables in TPB lack detailed classification and are relatively general. This research mainly considers social research and divides subjective norms into two categories. The conclusion has a more specific guiding role for decision-makers, manufacturers, and consumers.

After literature review, there are two research gaps as follows. First of all, although it is very urgent to understand the purchase and use factors of disposable products in the context of Chinese culture, compared with developed countries, less attention has been paid to this topic, and more articles are needed to deepen the discussion. Second, although the independent effects of these psychological factors, such as subjective norms and emotion, have been previously studied, how they interact with each other to influence single-use plastic products has not been sufficiently discussed. This study aims to analyze the interaction between subjective norms and positive anticipated emotions to explain the intentions to purchase single-use plastic products. In order to solve the knowledge gap, this paper studies the interaction between subjective norms and positive anticipated emotions on purchase intentions by analyzing the results of a questionnaire survey. Finally, the findings and policy implications will be discussed.

## 2. Theoretical framework and hypotheses

### 2.1. Literature review

Existing literature on the reduction of single-use plastic consumption behavior is mainly carried out from two perspectives: policy-oriented perspective and psychological-oriented perspective.

The most common policy-oriented perspective is bans on plastic items, most commonly plastic bags (Nielsen et al., 2019; Adeyanju et al., 2021). Wagner (2020) mentions policy approaches such as ‘command and control’ to reduce single-use plastic consumption in the context of expanded polystyrene tableware. Economic policies such as fees/taxes on particular items or financial incentives can promote the reduction of single-use plastic consumption behavior (Thomas et al., 2016). However, many studies have shown that the effect of these measures is not as good as imagined, because the assumption of a rational person will be influenced by social factors such as habits and emotions (Rivers et al., 2017; Luo et al., 2021). Scholars from the perspective of psychological-oriented perspective believe that the consumption behavior of disposable plastic products is affected by psychological factors such as attitudes, emotions, and environmental awareness (Sun et al., 2017; Heidbreder et al., 2019). They believe that it is effective to achieve the ultimate goal of sustainable development by emphasizing some psychological factors (such as attitude, convenience, social norms and reducing the inertia of purchasing plastic products). This study believes that disposable plastic consumption is caused by consumers’ daily behaviors, so it seems more reasonable to study disposable plastic consumption behavior from the perspective of psychological orientation.

In developing countries, especially in the context of China, there is a greater urgency to reduce single-use plastic products, and there are more and more studies focusing on such behaviors, but still relatively few. For example, Sun et al. (2017)’s study is mainly based on the theory of planned behavior, adding three variables of convenience, environmental concern, and ethical belief to discuss the willingness to use plastic bags.

Based on the above-mentioned literature review, understanding the purchase and use factors of single-use plastic products can better reduce such behaviors in a targeted manner, especially in the Chinese cultural background, but compared with developed countries, the attention paid to this topic is still less. More articles are needed to deepen the discussion. Second, while the independent effects of these psychological factors on subjective norms and emotion have been studied previously, how they interact with each other to influence single-use plastics has been largely ignored. In fact, existing literature has demonstrated the interaction between psychological factors in some other pro-environmental theme situations (e.g., Pham et al., 2021). However, psychological factors, especially the interaction of emotional and social factors, have not been sufficiently explored in the context of reducing single-use plastics. Table 1 shows selected studies on single-use plastic consumption.

**Table 1:** Selected studies on single-use plastic consumption.

Title	Country	Key findings	Author (Year)	
The synergistic impact of motivations on sustained pro-environmental consumer behaviors: an empirical evidence for single-use plastic products	Vietnam	Intrinsic and prosocial motivations are found to be significant predictors of sustained PECB.	Pham et al. (2021)	Psychological-oriented perspective
Understanding consumers’ intention to use plastic bags: using an extended theory of planned behavior model	China	The extension is implemented by adding three variables: convenience, environmental concern and ethical belief.	Sun et al. (2017)	
Why Do Consumers Switch to Biodegradable Plastic Consumption? The Effect of Push, Pull, and Mooring on the Plastic Consumption Intention of Young Consumers	China	This study investigates the push factors, including environmental threats, knowledge, and the strict regulative environment; pull factors, including alternative attractiveness and normative environment; and mooring factors, such as cost switching and self-efficacy.	Gao and Shao (2022)	
The Welsh single-use carrier bag charge and behavioral spillover	Welsh	A Single-Use Carrier Bag Charge (SUCBC) requires bags to be sold for a small fee, instead of free of charge.	Thomas et al. (2016)	Policy-oriented perspective
Policy instruments to reduce consumption of expanded polystyrene food service ware in the USA	USA	Reducing single-use plastic shopping bags in the USA	Wagner (2020)	
Plastic bag usage and the policies: A case study of China.	China	Results show a boomerang effect of the pricing policy (i.e., charging for plastic carrier bags) in China.	Wang and Li (2021)	
Tackling the plastic problem: a review on perceptions, behaviors, and interventions.	None	Economic policies such as fees, levies, and taxes were the more common policy instrument to reduce the use of single-use plastics.	Heidbreder et al. (2019)	
Effectiveness of intervention on behavior change against use of non-biodegradable plastic bags: a systematic review.	None	A combination of bans and fees/taxes was more common than either policy alone.	Adeyanju et al. (2021)	

### 2.2. Basic variables in TPB

TPB is one of the most frequently cited models used to understand the determinants of individuals’ social behaviors (Ajzen, 1991). TPB is frequently used for researches on green consumer behavior, such as organic food purchase behavior (Vabo and Hansen, 2016; Ashraf et al., 2018) and green purchase behavior (Ruangkanjanases et al., 2020; Zaremohzzabieh et al., 2020; Zhang and Luo, 2021). Attitude toward behavior, perceived behavioral control, and subjective norms determine intention, and intention directly determines behavior according to TPB (Ajzen, 1991). Attitude means individuals’ evaluation of certain behaviors, which can be positive or negative (Yadav and Pathak, 2016; Sun et al., 2021). Individuals’ intentions to participate the certain behaviors when they have positive attitudes toward behaviors, and vice versa. Perceived behavioral control means the perceived degree of difficulty of conducting certain behaviors (Wang et al., 2018; Dangelico et al., 2021). Individuals are willing to perform actions they consider easy to perform. Behavioral intentions also depend on perceived behavioral control (PBC), reflecting the degree to which individuals feel that it is easy or difficult to perform behaviors under given conditions.

Based on TPB, in the context of purchasing single-use plastic products, it can be inferred that if consumers have positive attitudes, a sufficient level of control (i.e., sufficient ability, ample time, and available opportunities), they will purchase single-use plastic products. It is easy to implement the behavior of purchasing single-use plastic products, and consumers’ intentions will increase. In this study, attitudes and perceived behavioral control were regarded as positive predictors of consumers’ intentions to purchase single-use plastic products. Therefore, we assume:

*H1*: Attitude positively influences intentions to purchase single-use plastic products.

*H2*: Perceived behavioral control (PBC) positively influences intentions to purchase single-use plastic products.

Subjective norms are regarded as the third influencing factor of behavioral intention. It is also a very important factor in Chinese collectivist culture. Consumers are social people, and their consumption behavior is largely influenced by other people (Sun and Wang, 2019). Individuals are always embedded in the social network, and individuals’ green consumption behavior decisions will inevitably be influenced by the social network. Researches have shown that the behavioral decisions of a certain subject will be affected by the behavior of other subjects in the group (Dangelico et al., 2021). Similarly, when individual consumers make decisions about green consumption behavior, they will not only be affected by factors such as their own psychology, cognition, and product attributes, but will also be largely affected by other individuals (or entire groups) in the network.

Within the framework of the TPB, subjective norms are the individuals perceive the social pressure of important others, which in some way have significant impact on consumers’ behavior (Sun and Wang, 2019; Dangelico et al., 2021). Subjective norms indicate that individuals are willing to follow the expectations or opinions of important others (i.e., family, relatives, or friends). Based on previous literature, we subdivide the subjective norms into normative social influence (NSI) and informational social influence (ISI) (Burnkrant and Alain, 1975; Wang et al., 2020). Normative social influence means the tendency of individuals to obey the expectations of others (Ru et al., 2019). It means normative social influence is the behavior that needs to be consistent with the expectations of important people and expected to be loved or accepted by important people. Consumers are more willing to purchase the same commodity with the influential others. Informational social influence refers to that individuals who tend to accord with the opinions or suggestions of important others in view of the information they get (Kuan et al., 2014). Thus, if the important reference personnel around the consumer talk about the damage to the environment caused by the white pollution caused by takeaway, the consumer’s intention not to purchase single-use plastic products will increase (Chen et al., 2018).

Refusing to purchase single-use plastic products is beneficial to environmental protection and has obvious externalities. In the context of single-use plastic products, it can be inferred that if the consumers consider important others (such as friends or relatives) advise them to reduce the purchase behaviors, their intentions not to purchase single-use plastic products will increase. In this study, NSI and ISI were seen as positive predictors of purchase intentions of single-use plastic products.

Therefore, we assume that:

*H3*: Normative social influence positively affects consumers’ intentions to purchase single-use plastic products.

*H4*: Informational social influence positively affects consumers’ intentions to purchase single-use plastic products.

### 2.3. Emotional factors

Although TPB has a good predictive ability for pro-environment behavior, TPB theory believes that individual behavior largely depends on the rational choice of individual cognitive factors. According to Kals et al. (1999), in a sense, an individual’s pro-environmental behavior cannot be regarded as the result of a completely rational choice. Many behaviors are also influenced by individual positive anticipated emotions, that is non-cognitive emotional factors like personal emotions, also play an important role in them. Emotions are feelings (such as negative or positive) to events or problems (Russell et al., 2017). A person is more likely to be involved in an event when he expresses positive expectations about the event or issue, otherwise, he is more likely to not be involved or care about the event. Positive anticipated emotion means the positive mental state in the implementation of a certain behavior (such as anti-plastic product purchase behavior) in this study. Han and Hyun (2018) pointed out that positive anticipated emotions include expected pride, excitement, and self-confidence, and they first indicate the importance of a problem or event, and thus provide stimulation for behavior. People know that positive expectations play an important role in the implementation of pro-environmental behaviors based on the theory of interpersonal behavior (Russell et al., 2017). Previous research has shown that there is a certain relationship between positive expectations and pro-environmental behavior (Webb et al., 2013). Thus, in the context of purchasing single-use plastic products, it can be inferred that when consumers think that it is good for the environment not to purchase single-use plastic products in daily life, and show positive anticipated emotion toward anti-plastic products, they are more inclined to not purchase single-use plastic products. Moreover, affective events theory also confirms our conjecture. The theory believes that positive anticipated emotion provides motivation for behaviors, and thus directly affects actual behavior (Han and Hyun, 2018; Wang et al., 2018). Therefore, this study hypothesizes that positive anticipated emotion directly influences on household anti-plastic plastic product purchase behavior, and we assume that consumers with higher positive anticipated emotion tend to not carry out actual purchase behaviors of single-use plastic products.

*H5*: Positive anticipated emotion negatively influences consumers’ intentions to single-use plastic products.

### 2.4. Interaction effect of subjective norms and emotion

Environmental behaviors, such as choosing single-use plastic products, have positive externalities, so they will be influenced by society. Personal important relationships (such as relatives and friends) are the main source of this social influence (Thomas and Sharp, 2013). Subjective norms reflect the personal perception of these important relationships that people think they should or should not perform specific behaviors. And this subjective norm will be affected by emotions, because social constructivism puts emotions and other psychological phenomena in social relations to investigate, and believes that emotions are the product of interpersonal interaction (Armon-Jones, 1986; Sui and Li, 2020). Thus, emotions can help people coordinate interpersonal communication and maintain good social relationships by providing information about others. Therefore, subjective norms may interact with emotions, and their interaction effects may affect behavioral intentions. In this study, we divide subjective norms into NSI and ISI (Burnkrant and Alain, 1975; Wang et al., 2020). Normative social influence is the behavior that needs to be consistent with the expectations of important people and expected to be loved or accepted by important people (Li, 2013). People accept the influence of people they think are important to them as evidence of consistency with other people’s beliefs, and this degree of consistency will be more pronounced when individuals have positive anticipated emotions. If people can realize that important people are participating in this environmental behavior without purchasing single-use plastic products, and when consumers have higher positive anticipated emotions, people will more voluntarily accept the influence of others through a sense of identity to maintain a good relationship with others. On the contrary, if an individual realizes that important persons are participating in nonenvironmental behaviors, but he has a positive anticipated emotion of environmental behaviors, then this emotion will increase his/her intention to adopt environmental behaviors and eventually weaken his/her intention to behave in line with important relationships. Thus, when an individual is affirmed by an important person, his/her positive anticipated emotions will make him/her more willing to choose anti-plastic products behavioral decisions that are inconsistent with others.

Informational social influence means that individuals tend to accord with the opinions of others based on the information they get (Nolan et al., 2008). When an individual receives anti-environmental information from an important person, the self-confidence and excitement generated by positive anticipated emotions will make consumers more self-confident and believe in their own decisions. This positive anticipated emotion will strengthen the concept of self and weaken the received information from others. Therefore, positive anticipated emotions will negatively moderate the informational social influence to purchase intentions of single-use plastic products.

*H6*: The interaction of normative social influence and positive anticipated emotion will negatively influence purchase intentions of single-use plastic products.

*H7*: The interaction of informational social influence and positive anticipated emotion will negatively influence purchase intentions of single-use plastic products.

**Figure 1:**
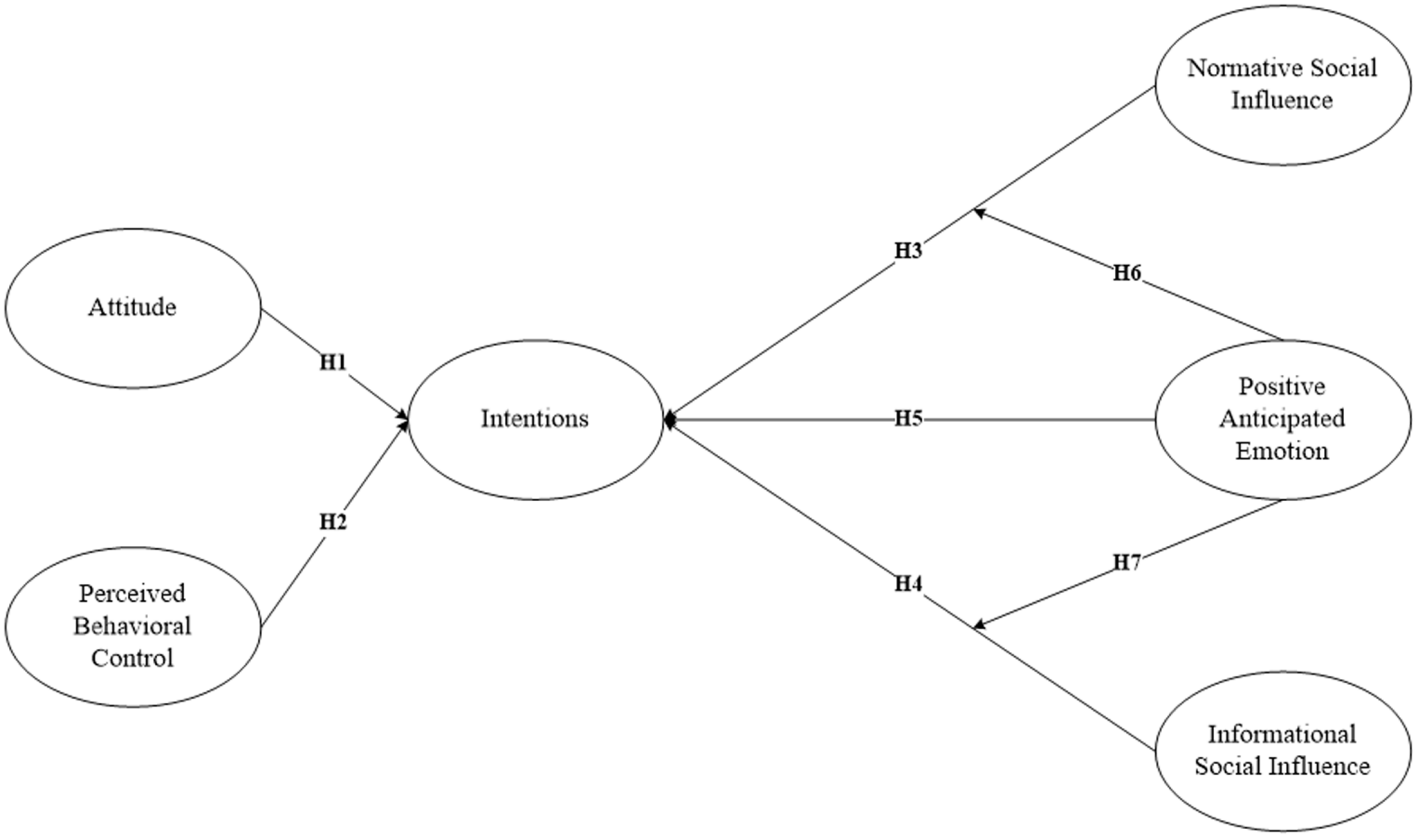
Research framework.

The research framework shown in Figure 1.

## 3. Methodology

### 3.1. Sample and data collection

The research team used questionaries survey to collect data online because of the epidemic. The questionnaire is published on “Survey Star” survey website, and the collection period is from mid-September to mid-October 2020 in China. The website has a powerful sample library, and the sample requirements can be specified according to the research theme and background when conducting research. Therefore, more data can be collected from different groups and individuals, making the sample more representative. The researchers conducted a random survey from the list of members of the “Survey Star” and finally extracted 600 samples. By forwarding the questionnaire link online, it is distributed randomly to consumers across the country. This not only expands the scope of personnel distribution of the questionnaire, but also collects information on personnel from different social classes, improving the universality and representativeness of the sample data. The participants who studied this survey were Chinese citizens over the age of 18 and all had supermarket shopping experience. This sample is similar to previous research samples on related topics, which also shows that the sample is representative (Sun et al., 2017; Luo et al., 2021). In order to avoid data discrepancies, respondents were told that the answers were anonymous and strictly confidential. In addition, there was no standard answer. In order to increase the response rate, respondents were told that they would receive 40 yuan as a reward after completing the questionnaire survey. In order to improve the response rate of the questionnaire, respondents will receive 40 yuan as a reward when they complete the questionnaires.

In the end, the researchers received a total of 566 completed questionnaires, and then eliminated singular values and missing values. There were 402 valid questionnaires in the end, and the recovery rate was 71.02% (402/566). The demographics of the respondents in this study are shown in Table 2.

**Table 2:** Demographic data of respondents.

Demographics	Frequency	Percentage (%)
Gender		
1. Male	199	49.50%
2. Female	203	50.50%
Age		
1. 18–30	299	74.38%
2. 31–40	44	10.95%
3. 41–50	42	10.45%
4. 51 and over	17	4.23%
Education level		
1. Senior middle school	25	6.22%
2. Junior college or university	245	60.95%
3. Master’s degree or above	132	32.84%
Income (average monthly)		
1. Less than ¥4,000 ($625)	143	35.57%
2.¥4,000–¥8,000 ($625–$1,250)	137	34.08%
3. ¥8,000–¥12,000 ($1,250–$1,876)	63	15.67%
4. More than ¥12,000 ($1,876)	59	14.68%
Total	402	100%

Furthermore, a t-test was performed to test potential no-response bias. Research team take the samples that return the complete questionnaire within 10 days as the early responses and the samples that return the complete questionnaire within the past 10 days as the late responses (Armstrong and Overton, 1977), and conduct a t-test to compare early responses and late responses. The results showed that the responses of the two groups were not significantly different at the 0.05 level. Therefore, it can be inferred that there is no main problem of non-response bias.

### 3.2. Questionnaire design and measures

Data collection using self-reported questionnaires. After completing the questionnaire design, the research team interviewed 7 scholars who opposed the purchase of single-use plastic products to solicit opinions on the questionnaire. Some minor changes were made to the questionnaire based on their feedbacks. Subsequently, the research team distributed 40 questionnaires for pilot surveys. According to the suggestions and feedback of the pilot survey, the questionnaire was revised and improved to facilitate the follow-up survey.

In this study, a multi-item scale measures the constructs because the constructs are latent variables. All items in the scale are adapted from previous researches. Some items are modified to adapt to the background of the purchase of single-use plastic products in this study. The research team used the Likert 7-point scale to measure all the items, and the respondents asked to score the items, ranging from “very disagree” to “very disagree.” The measurement scale of attitude, perceived behavioral control, and intentions to purchase single-use plastic products contained seven items were drawn from the research of Kim et al. (2013), Chen and Tung (2014), and Sun et al. (2017). The measurement scale of NSI and ISI contained six items was drawn from the research of Burnkrant and Alain (1975) and Wang et al. (2020). The measurement scale of positive anticipated emotion contained three items was drawn from the research of Cheung et al. (2017), Nilsson et al. (2014), and Wang et al. (2018). See Appendix A for a detailed introduction to the constructs and items.

## 4. Data analysis and results

### 4.1. Test of common method bias and normal distribution

In structural model analysis, the anonymity and hints of the questionnaire survey can alleviate the common method bias (CMB), but there is still a need to further evaluate the CMB. Harman’s single-factor test was carried out to evaluate the CMB (Harman, 1976). The results show that the first factor explained only 39.6% of the variance, less than 50.0% of the baseline (Harman, 1976). The results show that CMB is not the main focus of this study. In addition, in order to ensure that the structural equation model (SEM) assumptions are met, a normal distribution test is required before testing the SEM. As shown in Table 3, the absolute values of Skewness are less than 3, and the absolute values of Kurtosis are less than 10, indicating that the data deviates from the normal distribution insignificantly (Kline, 1998). The VIF values were all less than 10, indicating that none of the variables in Table 3 exhibited multicollinearity (Yan et al., 2022).

**Table 3:** Results of confirmatory factor analysis.

Construct	Items	Loading	Composite reliability	Cronbach’s α	AVE	Skewness	Kurtosis
Attitude (ATT)	ATT1	0.90^***^	0.87		0.70		
	ATT2	0.71^***^		0.88		0.143	−0.68
	ATT3	0.87^***^					
Perceived Behavioral Control (PBC)	PBC1	0.82^***^	0.82		0.60		
	PBC2	079^***^		0.82		−0.405	−0.400
	PBC3	0.72^***^					
Normative Social Influence (NSI)	NSI1	0.89^***^	0.87		0.69		
	NSI2	0.93^***^		0.85		−0.119	−0.398
	NSI3	0.64^***^					
Informational Social Influence (ISI)	ISI1	0.83^***^	0.83		0.61		
	ISI2	0.64^***^		0.82		0.044	−0.455
	ISI3	0.86^***^					
Positive Anticipated Emotion (EM)	EM1	0.73^***^	0.80		0.58		
	EM2	0.80^***^		0.80		−0.379	−0.582
	EM3	0.75^***^					
Intention to purchase single-use plastic products (INT)	INT1	0.89^***^	0.86		0.67		
	INT2	0.82^***^		0.86		0.023	−0.767
	INT3	0.75^***^					

^***^p < 0.001.

### 4.2. Measurement model analysis

Confirmatory factor analysis (CFA) is used to indicate the measurement characteristics of each construct, such as reliability and validity. The CFA results show that the measurement model is acceptable. The index of model fitting is as follows: *χ*^2^/df is 2.907, CFI is 0.95, IFI is 0.95, TLI is 0.93, and RMSEA is 0.069. To test the reliability of the constructs, composite reliability was explored. In Table 3, all the composite reliability ranges from 0.80 to 0.87, all the Cronbach’s α ranges from 0.80 to 0.88, which are higher than 0.70. According to Fornell and larcker (1981), the results support the reliability of the construct. To test the validity of the construct, convergent validity and discriminant validity were explored. The AVE and factor loadings were used to test the convergent validity. Table 4 shows AVE is higher than 0.50, and most of the factor loadings are higher than 0.70 (Fornell and Larcker, 1981; Hair et al., 1998). The construct has good convergent validity. Tables 3, 4 show the construct has good discriminant validity since the correlation between constructs is less than the square root of AVE.

**Table 4:** Mean, standard deviation, and correlation.

Construct	Mean	SD	ATT	PBC	NSI	ISI	EM	INT
ATT	3.32	1.47	**0.83**					
PBC	4.50	1.40	0.30^**^	**0.78**				
NSI	3.55	1.29	0.52^**^	0.35^**^	**0.83**			
ISI	3.44	1.33	0.37^**^	0.26^**^	0.53^**^	**0.78**		
EM	4.28	1.33	−0.28^**^	−0.21^**^	−0.36^**^	−0.21^**^	**0.76**	
INT	3.63	1.56	0.57^**^	0.44^**^	0.73^**^	0.42^**^	−0.46^**^	**0.82**

(1) Bold elements are the square root result of AVE.

(2)^**^p < 0.01.

Likewise, the evaluation method of HTMT (Table 5) is based on inferential statistics and uses confidence intervals to measure discriminant validity. HTMTs are associated with dissipative construct scores for assessing relationships between constructs. This study further shows that there is no issue of discriminative validity based on a < 0.9 threshold (Yan et al., 2022).

**Table 5:** Discriminant validity of first-order construct using Heterotrait–Monotrait Ratio (HTMT).

Construct	ATT	PBC	NSI	ISI	EM	INT	Tolerance	VIF	*R*^2^
ATT							0.697	1.435	0.32
PBC	0.359						0.847	1.180	0.19
NSI	0.604	0.423					0.551	1.816	0.54
ISI	0.441	0.316	0.633				0.707	1.414	0.18
EM	−0.339	−0.254	−0.440	−0.265			0.697	1.435	0.21
INT	0.656	0.524	−0.861	0.510	−0.549				0.64

### 4.3. Structural model and hypothesis analysis

The structural model is analyzed to verify the research hypotheses. In general, the CFA results show that the structural model is acceptable. The index of model fitting is as follows: χ^2^/df is 2.907, CFI is 0.91, IFI is 0.95, TLI is 0.93, and RMSEA is 0.07. The results of structural model analysis are shown in Table 6. As expected, the effects of attitude (*β* = 0.230, *t* = 6.72, *p* < 0.001), perceived behavioral control (*β* = 0.230, *t* = 4.88, *p* < 0.001), normative social influence (*β* = 0.290, *t* = 5.82, *p* < 0.001), informational social influence (*β* = 0.190, *t* = 3.960, *p* < 0.001) on intention to purchase single-use plastic products. In addition, the effects of positive anticipated emotion (*β* = −0.190, *t* = −3.470, *p* < 0.001) on intention to purchase single-use plastic products. The results show that all hypotheses are verified, except for H6.

**Table 6:** Path coefficients of the structural model.

Path	Path coefficient	*T*-value	Hypothesis	Results
ATT → INT	0.23	6.72^***^	H1	Supported
PBC → INT	0.23	4.88^***^	H2	Supported
NSI → INT	0.29	5.82^***^	H3	Supported
ISI → INT	0.19	3.96^***^	H4	Supported
EM → INT	−0.19	−3.47^***^	H5	Supported
NSI*EM → INT	0.21	3.42^***^	H6	N.S.
ISI*EM → INT	−0.26	−4.17^***^	H7	Supported

^***^p < 0.001.

**Figure 2:**
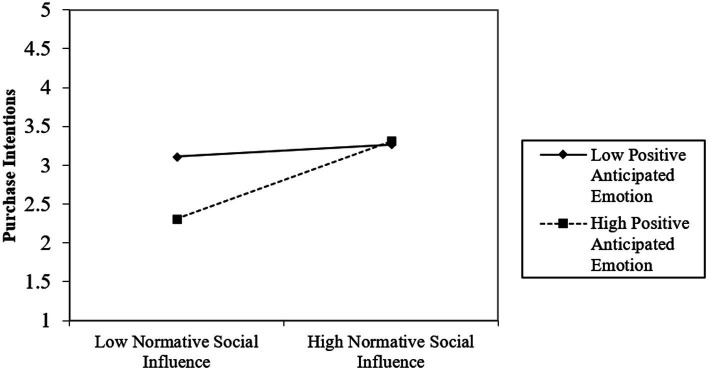
The moderating effect of positive anticipated emotion on the relationship between normative social influence and purchase intention of single-use plastic products.

**Figure 3:**
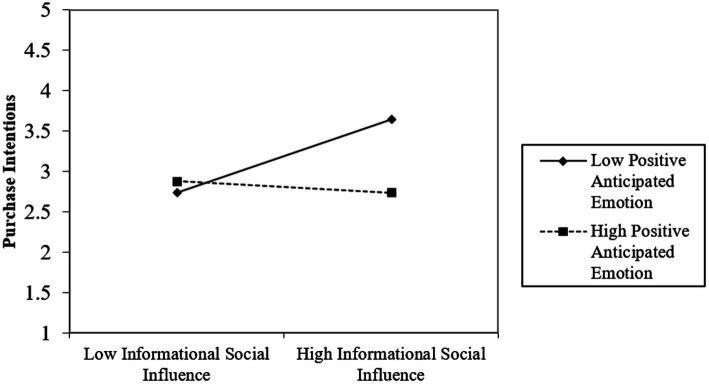
The moderating effect of positive anticipated emotion on the relationship between informational social influence and purchase intention of single-use plastic products.

The interaction term of NSI × EM (*β* = 0.210, *t* = 3.420, *p* < 0.001) positively affects intention to purchase single-use plastic products. The results showed that EM positively moderated the relationship between NSI and purchase intention. The moderating effect is shown in Figure 2, showing the different strengths of the relationship between NSI and purchase intention when EM levels are one SD above and one SD below their mean. The interaction term of ISI× EM (*β* = −0.260, *t* = −4.170, *p* < 0.001) negatively affects intention to purchase single-use plastic products. As shown in Figure 3, when EM was one SD above its mean, the effect of ISI was weaker than when ISI was one SD below its mean. These results indicate that H7 is supported but H6 is not. Figures 2, 3 show the slope analyses for the two significant interaction terms.

Several control variables (i.e., gender, education, income, and age) are also considered in this research. Gender (*b* = −0.19, *t* = −1.88, *p* > 0.05), education (*b* = 0.02, *t* = 0.20, *p* > 0.05), and income (*b* = 0.02, *t* = 0.40, *p* > 0.05) have no significant effect on consumers’ intentions to purchase single-use plastic products, while age (*b* = −0.14, *t* = −2.91, *p* < 0.01) has a significant negative effect on purchase intention of single-use plastic products. Furthermore, the extended TPB model (64.1%) has an 8% (∆*R*^2^ = 10.1%, *p* < 0.01) increase in interpretation over the original model (54.0%). Thus, we can infer that this model has a better predictive effect.

## 5. Discussion and implication

### 5.1. Discussion

Theoretically, this study proposes a model combines cognitive factors, especially social cognitive factors and emotional factors, thus enriching and contributing to the literature on single-use plastic products purchase. Previous studies neglected to explore the role of emotional factors and interaction with social cognitive factors (Cheung et al., 2017; Sun et al., 2017). This study enriches the emotional and social cognitive factors in the purchase of single-use plastic products and highlights the importance of emotional and social cognitive interactions.

As expected, the findings presented those attitudes to purchase single-use plastic products, perceived behavioral control, NSI, and ISI positively influence consumers’ intentions to purchase single-use plastic products, while positive anticipated emotion negatively affects theirs’ intentions. The results are consistent with previous researches (Sun et al., 2017; Wang et al., 2018). It can be speculated that people with negative attitude, lower perceived behavioral control, lower NSI, lower ISI, and higher positive anticipated emotion prefer to form intentions not to purchase single-use plastic products.

In addition, the results show that normative social influence has the greatest impact on individuals’ intentions to purchase single-use plastic products. The results are consistent with previous researches (Ru et al., 2019; Wang et al., 2020). It may be because individuals are more willing to belong to specific group and avoid being isolated by others and imitating the behavior of others in the group. This is especially true in China where collectivist culture prevails (Sun and Wang, 2019). Besides, most people tend to take a wait-and-see attitude and are unwilling to execute the behavior first. Therefore, if their leaders and colleagues take the action of not purchasing single-use plastic products, individuals will also not purchase single-use plastic products. This finding confirms that normative social influence is effective in predicting pro-environmental behaviors in earlier studies.

The results show that informational social influence positively affects individuals’ intentions to purchase single-use plastic products. The results are consistent with previous researches (Chen et al., 2018; Wang et al., 2020). It may be because informational social influence means the fact that people tend to use the information they obtain from important relatives as evidence of behavioral decision-making. Family and friends are an important and credible source of information for individuals in choosing products and related alternative products or services. If important reference persons around the consumer talk about the good experience brought by a certain product and advise them to buy the product or service, then consumers’ purchase intentions for the product will increase. With the issuance of the ban on plastics, consumers began to realize the irrationality of purchasing single-use plastic products. Moreover, this kind of information about not purchasing single-use plastic products has spread among consumers. Therefore, the information about not purchasing single-use plastic products by family and friends will significantly affect the personal behavior of purchasing single-use plastic products.

The research results also show that consumers’ positive anticipated emotions will significantly influence consumers’ intentions to purchase single-use plastic products. The results are consistent with previous researches (Han and Hyun, 2018; Wang et al., 2018). It may be because in their daily lives, when consumers think that buying and using single-use plastic products is harmful to the environment and show negative expectations about buying and using single-use plastic products, they are more inclined not to buy single-use plastic products. This finding is also consistent with the emotional event theory, which believes that positive anticipated emotions directly predict actual behavior, because it is the driving force that stimulates individual action (Han and Hyun, 2018). Therefore, positive anticipated emotions have reduced consumers’ behavior in purchasing and using single-use plastic products.

The results indicate that positive anticipated emotion negatively moderates the relationship between ISI and single-use plastic product purchase behavior. The results are consistent with previous researches (Wang et al., 2020). This may be because positive anticipated emotions will increase the mood of green behaviors (Russell et al., 2017; Wang et al., 2019; Dangelico et al., 2021). Positive anticipated emotions will not immediately affect the behavior although they are non-cognitive factors, because informational social influence has a long-term impact on people. Thus, positive anticipated emotions will gradually deepen as consumers think about the impact of the information society, and then enhance the personal environmental behavior without purchase single-use plastic products. However, the results also show that if most people who are important to them purchase single-use plastic products, then they prefer to buy and purchase single-use plastic products, and this relationship is positively moderated by positive anticipated emotions. The results are not consistent with previous researches (Li, 2013). This is because positive anticipated emotions will make people more willing to join the group, and they will regard themselves as part of the group. And unlike cognitive factors, affective factors act more directly on current behaviors. Positive anticipated emotions, as a non-cognitive factor, directly confidence in integrating into the group currently, but ignore the positive externalities of the behavior itself. Thus, if most people who are important to them advocate and encourage them to purchase single-use plastic products, then they are more willing to buy and purchase single-use plastic products, even if the behavior itself does not have a positive externality.

### 5.2. Theoretical and managerial implications

#### 5.2.1. Theoretical implications

First, understanding the factors behind the purchase and use of single-use plastic products in developing countries such as China is urgent, and more articles are needed to explore them in depth. This study is very timely and could enrich the relevant literature.

Second, while the independent effects of psychological factors (such as subjective norms and emotions) have been studied previously, how they interact to affect single-use plastic products has not been fully discussed. This study first considered emotional factors on the basis of cognitive factors, and considered the interaction with emotional factors in the context of social research, which enriched the application of the theory of planned behavior in the context of purchasing disposable plastic products. Incorporating affective factors and their interactions with subjective norms into TPB to understand consumers’ environmental protection behaviors enriches the understanding of consumers’ behavior of not buying single-use plastic products.

Finally, the rationality of TPB in the field of green behavior is verified. It has been suggested that attitudes, subjective norms, and perceived behavioral control are directly proportional to consumers’ intentions. However, the three variables in TPB lack detailed classification and are rather general. Considering primarily social research, this study divides subjective norms into two categories. We look forward to more specific and detailed follow-up related research.

#### 5.2.2. Theoretical implications

Theoretical research shows that there are many factors that affect consumers’ purchase behaviors of single-use plastic products. However, in terms of management, this research will help companies and government authorities design plans to reduce the use behaviors of single-use plastic products and increase the reuse behavioral intentions of single-use plastic products. The five structures that affect consumer intentions are attitude, perceived behavioral control, NSI, ISI, and positive anticipated emotion. In addition, NSI, ISI, and positive anticipated emotion also interact with purchase intentions of single-use plastic products.

The results show that normative social influence is the main reason for individuals to purchase single-use plastic products. Many consumers see that their family and friends do not purchase single-use plastic products, so they will also not join in this behavior. Therefore, public authorities may position opposition to the purchase behaviors of single-use plastic products as a social trend, showing consumers the frequency or percentage of the local population not purchasing single-use plastic products. Campaigns against single-use plastic products can also be held to illustrate the environmental hazards of single-use plastic products, and to encourage celebrities, Internet influencers, and family members to take the lead in resisting white pollution. The results also show that informational social influence is also the main factor that affects the purchase behaviors of single-use plastic products by individuals. Relatives and friends are a more trusted source of information. If individuals find that most people who are important to them advocate and encourage them not to purchase single-use plastic products, they will actively participate in activities that oppose the purchase behaviors of single-use plastic products. Public authorities should actively educate consumers about the adverse effects of plastic product purchase on the environment, inform consumers of the important impact of their individual behaviors on others, and encourage consumers to spread the adverse effects of white pollution on the environment to their family and friends through their own efforts.

This study also found that positive anticipated emotions effectively hindered the spread and purchase behaviors of single-use plastic products. In addition, emotional factors are also the positive factors inducing consumers’ intentions not to purchase single-use plastic products. The results show that when consumers are satisfied, excited, and happy, they are more willing to refuse single-use plastic products. Therefore, researchers and practitioners dealing with emotions must realize that strengthening positive anticipated emotions is essential for consumers to purchase single-use plastic products. In order to encourage consumers to generate positive anticipated emotions, sellers can paint beautiful pictures on some paper alternative packaging, and design the language to thank consumers for being environmentally friendly. At the same time, government could provide consumers with reports to emphasize the positive consequences of the anti-purchase of single-use plastic products and explain benefits of using paper products and reusing single-use plastic products to cultivate their positive expectations about not purchasing single-use plastic products (Fornara et al., 2016). For example, tell consumers that by resisting the purchase behaviors of single-use plastic products can reduce carbon emissions for the world, reduce the energy crisis, and reduce white pollution.

The interaction between NSI and ISI and positive anticipated emotions presents a completely opposite relationship to consumers’ purchase behaviors of single-use plastic products. Therefore, public authorities should understand the importance of emotions to different consumers in order to tailor strategies to reduce the purchase behaviors of single-use plastic products accordingly. For a given level of normative social influence, consumers with higher positive anticipated emotions will be more willing to adopt single-use plastic products. Therefore, public authorities should remind consumers of the adverse effects of single-use plastic products on environmental pollution when shopping in groups, so as to reduce consumer emotions and thereby reduce consumers’ purchase behaviors of single-use plastic products. For a given level of informational social influence, consumers with higher positive anticipated emotions will be more opposed to purchasing single-use plastic products. Therefore, public authorities should encourage consumers to spread the environmental pollution behaviors of single-use plastic products through word of mouth through social media, channels, etc., praise the consumers for not purchasing single-use plastic products for environmental protection, and thank them for their efforts in environmental protection. Hence, these positive anticipated emotions will encourage consumers to resist the purchase behaviors of single-use plastic products.

## 6. Conclusions and limitations

### 6.1. Conclusion

The findings presented that attitude, perceived behavioral control, normative social influence, informational social influence have positive effects on consumers’ intentions to purchase single-use plastic products, while positive anticipated emotion negatively affects consumers’ intentions to purchase single-use plastic products. Positive anticipated emotion negatively moderates the relationship between NSI and consumers’ intentions to purchase single-use plastic products, but positively moderates the relationship between ISI and consumers’ intentions to purchase single-use plastic products.

### 6.2. Limitations

There are a few limitations in our research although it provides some enlightening results and implications. First of all, the research investigates consumers’ purchase intentions, not their actual purchase behavior. There is still a gap between behavioral intention and behavior although scholars generally believe that behavioral intention can directly predict behavior (Ajzen, 1991). Second, this research uses questionnaire surveys to collect data, which rely on respondents’ self-reports, which are cross-sectional data. The research results based on these data may not fully reflect the causal relationship. At the same time, there are limitations in the data collection method, and there may also be insufficient randomness in the sampling process. Since the samples of this research are mainly collected online, it restricts the participation of some consumers who are too old to fill out the questionnaire, which affects the universality of the sample to a certain extent. More data collection methods such as in-depth interviews and longitudinal design can be used in future research. Finally, other variables, such as personal norms and green knowledge, may also be decisive factors for consumers to purchase single-use plastic products. Accordingly, additional exploration should be carried out.

## Data availability statement

The raw data supporting the conclusions of this article will be made available by the authors, without undue reservation.

## Author contributions

YS and HH contributed to the research design. YS conducted the sample collection and data analysis and wrote the paper. HH also did text review and editing. All authors contributed to the article and approved the submitted version.

## Funding

This research was supported by the National Social Science Foundation of China [Grant number 22BGL093] and the Humanity and Social Science Youth Foundation of Ministry of Education of China [Grant number 22YJC630122].

## Conflict of interest

The authors declare that the research was conducted in the absence of any commercial or financial relationships that could be construed as a potential conflict of interest.

## Publisher’s note

All claims expressed in this article are solely those of the authors and do not necessarily represent those of their affiliated organizations, or those of the publisher, the editors and the reviewers. Any product that may be evaluated in this article, or claim that may be made by its manufacturer, is not guaranteed or endorsed by the publisher.

## Appendix A

Constructs and measurement items.

**Table:**

Construct	Items	Coding
**Predict variable:**		
Attitude toward using plastic bags (ATT)	It is a good idea to purchase single-use plastic products.	ATT1
	It is a wise choice to purchase single-use plastic products.	ATT2
	I think I should take action to reduce the purchase single-use plastic products.	ATT3
Perceived behavior control (PBC)	Single-use plastic products are generally available in the shops while shopping.	PBC1
	It is convenient for me to purchase single-use plastic products.	PBC2
	I can purchase single-use plastic products easily if I want to.	PBC3
Normative social influence (NSI)	I usually identify with other people by purchasing single-use plastic products like everyone else.	NSI1
	I gain a sense of belonging by purchasing single-use plastic products like others.	NSI2
	I often try to act like others if I want to be like them.	NSI3
Informational social influence (ISI)	I frequently ask my friends about the purchase single-use plastic products.	ISI1
	I frequently consult my family and friends about the purchase single-use plastic products.	ISI2
	I often collect information about the purchase single-use plastic products from friends and family.	ISI3
**Moderator variable:**		
Positive anticipated emotion (EM)	I will feel excited if I refuse to purchase single-use plastic products.	EM1
	I will feel relaxed if I refuse to purchase single-use plastic products	EM2
	I will feel proud if I refuse to purchase single-use plastic products.	EM3
**Predictor variable:**		
Intention to purchase single-use plastic products (INT)	I intend to purchase single-use plastic products.	INT1
	I prefer to choose purchase single-use plastic products.	INT2
	I am willing to purchase single-use plastic products.	INT3
